# Editorial for the Glassy Materials and Micro/Nano Devices Section

**DOI:** 10.3390/mi16020117

**Published:** 2025-01-21

**Authors:** Giancarlo C. Righini

**Affiliations:** Istituto di Fisica Applicata ‘Nello Carrara’ (IFAC), National Research Council (CNR), Sesto Fiorentino, Metropolitan, 50019 Florence, Italy; righini@ifac.cnr.it

Glass is an amorphous solid, renowned for its transparency and versatility, and has been widely used for centuries in both scientific instruments and daily life. Despite its long history, providing an accurate definition of glass has posed a challenge for scientists. Even today, there is no universally accepted answer to the question, “What is glass?” However, a relatively recent article gained significant consensus by proposing the following intuitive definition: “Glass is a nonequilibrium (*metastable*), non-crystalline state of matter that appears solid on a short time scale but continuously relaxes towards the liquid state” [1,2].

In addition to this fundamental aspect, there are numerous important research and development areas focused on the properties of glass and its use in manufacturing objects for both everyday life and scientific instruments and devices. Glass has undoubtedly played a pivotal role in advancing science. Its unique physical and chemical properties have made it essential across a wide range of scientific fields, from optics to chemistry and beyond, and from large-scale objects like astronomical lenses to microdevices such as integrated optical chips. The critical role of glass in modern society has led to the designation of our era as “The Glass Age”, and in recognition of its significance, the United Nations declared 2022 the International Year of Glass (IYoG) [3].

Glass-like behavior is also exhibited by several materials sharing the amorphous structure of traditional glass [4]. This class includes a variety of substances, such as polymers, some composites, metals, and certain organic compounds. A key characteristic of these materials is their ability to transition from a supercooled liquid to a solid state without forming a crystalline lattice, a process known as the glass transition. Glassy materials also prove to be valuable in a wide range of technological, scientific, and industrial applications.

In recognizing the significance and relevance of glass and glassy materials, I am pleased to announce a new section, Glassy Materials and Micro/Nano Devices, in the journal of *Micromachines*. This section welcomes original research and review articles on either fundamental investigations or application-oriented research that can enlighten the recent advances in this field with regard to both materials’ characteristics and processing and manufacturing technologies.

The topics of interest include, but are not necessarily limited to, synthesis of glass and glassy materials, in either bulk or thin-film form; physical and chemical deposition processes; glass modeling; optical, spectroscopic, and structural characterization; conventional and laser processing of glass; fabrication of microstructures in glass and glassy materials; fabrication of microdevices using glass and glassy materials; and applications of these materials and microdevices.

For purely illustrative purposes and without aiming to be exhaustive, original and review articles are welcome that address any of the following issues.

## 1. Materials

Oxide and non-oxide glasses [5,6,7,8]. Glass is central to the construction of lenses, mirrors, and gratings used in microscopes, telescopes, and cameras in the visible, near-infrared, and infrared wavelength spectrum. Glass fibers have revolutionized global connectivity by enabling high-speed data transmission in telecommunications.

Glassy materials: amorphous polymers, such as polycarbonate and polymethyl methacrylate (PMMA), are widely used in optical lenses, lightweight windows, and shatter-resistant materials [9,10,11]. Glassy polymers are widely used as structural materials in aerospace vehicles, airplanes, automobiles, and biomedical devices [12]. They also play a role in advanced battery designs and solid-state electrolytes, enhancing energy storage efficiency.

Metallic glasses, i.e., amorphous metals, are a unique class of materials that combine the properties of metals and glasses [4,13,14]. Unlike crystalline metals, metallic glasses have a disordered atomic structure, resulting in exceptional properties: remarkable strength and elasticity, making them ideal for applications in aerospace and defense industries; corrosion resistance, which extends their longevity in harsh environments; and magnetic properties, which make them valuable in transformer cores and electronic components.

Metal–organic or hybrid glasses. As described in a very recent paper, the key concept of hybrid glasses deals with glasses that contain both inorganic and organic moieties linked together by a molecular bridging ligand [15]. Among other advantages, hybrid glasses could overcome the present difficulty of stably incorporating large molecules (e.g., inorganic or hybrid complexes, molecular catalysts, organic dyes, and enzymes) in a conventional glass, due to the needed high-temperature thermal processes. A pioneering step in this direction was related to the formation of organic–inorganic networks by sol–gel chemistry [16].

Glass-ceramics are inorganic, non-metallic materials prepared by controlled crystallization of glasses via different processing methods. They contain at least one type of functional crystalline phase and a residual glass [17,18,19,20]. They are produced by either top-down or bottom-up techniques, resulting in materials that exhibit enhanced mechanical, thermal, and optical properties compared to conventional glasses. This crystallization process allows for the fine-tuning of their microstructure, enabling precise control over their properties. Thanks to their versatile properties, glass-ceramics find applications in a wide range of industries, from aerospace and defense to optics and electronics to consumer goods and healthcare.

## 2. Material Processing and Microfabrication

Fabricating microdevices in glass presents significant challenges due to the material’s inherent physical and chemical properties. Key issues include the brittleness of glass, its high melting temperature, challenges associated with chemical etching, thermal expansion mismatches, and difficulties in surface functionalization. Even the process of cutting glass can be intricate; a recent study [21] compares conventional and laser cutting methods. Despite these challenges, glass remains a promising material for microdevice fabrication because of its exceptional properties, which make it suitable for a wide range of applications [22,23,24]. However, its hardness and brittleness complicate micro-level fabrication, often leading to extended machining times, high costs, and poor surface quality, particularly for smooth, high-aspect ratio structures [25]. Fortunately, advancements in micromanufacturing have enabled complex machining conditions and geometries, paving the way to harness the unique properties of glass at the micro-scale.

## 3. Microdevices and Applications

Glass microdevices are widely used in various fields, including microfluidics, bioengineering, optics, and chemical analysis, due to their chemical resistance, optical transparency, and ease of surface modification. Table 1 shows, as an example, a list of microdevices fabricated in glass or on glass substrates and their possible applications.

There are numerous other micro- and nano-scale devices that harness the unique properties of glass and glassy materials. In the following, I will focus briefly on optical and photonic devices. Referring to optical applications, a growing demand has emerged to integrate glass lenses into complex microsystems for high-tech products, particularly lighting devices. For example, many LEDs and laser diodes used in automotive applications require encapsulated microlenses. Consequently, the ability to manufacture glass microlenses cost-effectively at the wafer level using replication technology has become a critical focus [64]. Microlens arrays (MLAs), moreover, play a vital role in the development of compact imaging and focusing systems. Glass MLAs offer significant advantages in optical communication, sensing, and high-sensitivity imaging, thanks to their superior optical properties, mechanical durability, and physicochemical stability [65,66,67]. Recent innovations include large-scale fabrication of arrays (e.g., 700 × 700 microlenses) in chalcogenide glasses, which are essential for applications such as infrared imaging, non-contact thermography, night surveillance, night vision, and driver assistance systems [68,69,70].

Since the invention of lasers in the 1960s, photonics has grown into a field that permeates virtually every aspect of modern life, and photonic glasses (e.g., fiber glasses, laser glasses, nonlinear optical glasses, photochromic/photosensitive glasses, and magneto-optical glasses) have been a crucial ingredient of such a development [71,72]. In this field as well, microlenses may play an important role, e.g., to optimize the coupling efficiency between a semiconductor laser and a single-mode fiber [73]. At the distal end of a fiber, too, a microlens may be very useful to focus the radiation or to shape the intensity of the output light; in the latter case, conical tips may also be quite effective, e.g., to produce radiation patterns needed for medical applications [74,75]. The tip geometry can be simply and effectively created by thermally processing the fiber itself, producing the desired integral component.

Glass is an excellent material for many guided-wave components and circuits. The optical fiber is obviously the brightest example: ultrapure silica and specialty glass compositions have allowed us to fabricate single-mode conventional fibers exhibiting ultra-low losses as well as fibers with innovative geometries, such as microstructured or photonic-crystal fibers [76,77,78]. The introduction of microstructures, like gratings, into an optical fiber has proven to be very effective for sensing, offering unique characteristics of high sensitivity, high resolution, and excellent distributing and multiplexing capabilities [79]. Recent advances in nanofabrication technologies have enabled the development of a novel platform known as lab-on-fiber (LOF), which involves creating micro- and nanostructures on the fiber surface, either on the end facet or outer cladding. This breakthrough allows us to design and fabricate multifunctional, all-in-fiber devices that hold significant potential for applications not only in life sciences but also in the field of information and communication technology (ICT) [80,81]. The inclusion of nanoparticles in the fiber’s core may also open new perspectives for laser and sensing [82]. Doping fibers with rare earth ions has allowed us to develop high-quality, high-performance optical amplifiers and lasers [83,84].

In parallel with the development of optical fibers, integrated optics has grown and has attracted considerable attention [85], finding many applications in various areas, including quantum photonics [86]. A variety of passive or active waveguide devices are based on microstructures, which can be effectively realized by different technologies; direct laser writing and ion exchange are among the most popular, offering the advantage of producing monolithic glass circuits [87,88,89,90]. Planar microlenses and microlens arrays represent an effective solution for efficient coupling of lasers to integrated optical waveguides [91,92] and also for more complex circuits [93]. Excellent planar amplifiers and lasers can be easily fabricated in rare earth-doped glasses [89,90,94,95,96].

Besides fiber and integrated optics, other photonic structures allow us to confine light. In the last decades, much interest has been addressed to whispering gallery mode (WGM) microresonators, which are highly specialized optical devices that confine light through continuous internal reflection along their curved surfaces [97]. Named after the acoustical phenomenon observed in domed structures, WGMs enable light to circulate within a microresonator’s boundary, creating a resonant field. WGM microresonators come in shapes like spheres, bubbles, toroids, or disks and are characterized by their exceptional quality factor (Q-factor) and small mode volumes, enabling them to achieve high optical confinement and low energy loss. Their compact size and ability to support high-intensity light make them ideal for advanced photonic and sensing systems [98,99,100,101,102,103]. Glass-based photonic crystals (PhCs), which are spatially ordered structures with lattice parameters comparable to the wavelength of propagating light, have also proven to be excellent system for photon management. As an example, PhCs fabricated in a highly nonlinear chalcogenide glass are a promising platform for realizing compact all-optical switches operating at very low power and integrated in a chip [103]. 1D PhCs have been used to enhance rare earth luminescence thanks to the electric field confinement characteristics of these structures [104]. 3D photonic crystals, fabricated by sol–gel technology, demonstrated to be excellent mechanochromic sensors for a broad spectrum of applications [105].

## 4. Future Perspectives

I hope this brief and admittedly non-exhaustive summary of the state-of-the-art has provided a useful overview of the many applications that benefit from the unique characteristics and properties of microparts and microdevices made from glass and glassy materials. It is clear that there remains substantial room to explore new materials, innovative microtechnologies, and groundbreaking applications. Let me highlight a few promising and challenging topics that merit particular attention in the near future: gaining deeper insights into emerging materials, such as hybrid glasses [15] and glassy gels with remarkable adhesive, self-healing, and shape-memory properties [106]; advancing additive manufacturing techniques for glass and glassy materials; expanding the utilization of the unique properties of metallic glasses; and developing flexible and stretchable photonic microdevices leveraging the capabilities of flexible glasses.

## Figures and Tables

**Table 1:** Examples of microdevice systems made in glass and glassy materials and of their applications.

Topic	Microdevice	Application
Microfluidic chips [26,27,28,29,30,31,32]	Lab-on-a-chip systems	Chemical and biological analyses; sensing; drug delivery
	Droplet generators	Used in emulsification and single-cell analysis
	Microreactors	For high-precision chemical synthesis.
	Micropumps and valves	For fluid control in microfluidic systems.
Bioanalytical devices [33,34,35,36,37]	DNA microarrays	For genetic analysis and sequencing
	Protein microarrays	For studying protein interactions
	Electrophoresis chips	For separation and analysis of biomolecules like DNA and proteins
Chemical sensors [38,39,40,41,42]	pH sensors	Using functionalized glass surfaces
	Ion-selective electrodes	Chemical analysis in micro- and nanosystems integrated into glass microfluidic devices
	Gas sensors	For environmental monitoring
Medical devices [43,44,45,46,47]	Drug delivery systems, bioactive glass nanoparticles	Using microchannels to control the release rate. Cancer therapy
	Organ-on-a-chip devices	Simulating human organ systems for drug testing
	Blood plasma separators	For point-of-care diagnostics.
Energy devices [48,49,50,51,52]	Micro fuel cells	For compact power generation
	Micro heat exchangers	For thermal management.
	Photovoltaic microstructures	For solar energy applications.
Microelectromechanical systems (MEMS) [53,54,55,56,57,58,59,60,61,62,63]	Microsensors	Pressure, temperature, or humidity sensors
	Micromirrors	For optical switching and projection system
	Microtransformers, gyroscopes, microshutters	For RF-IC applications, automotive, control of light transmission
